# Facilitating translational team science: The project leader model

**DOI:** 10.1017/cts.2019.398

**Published:** 2019-08-28

**Authors:** Lynn Sutton, Lisa G. Berdan, Jean Bolte, Robert M. Califf, Geoffrey S. Ginsburg, Jennifer S. Li, Jonathan McCall, Rebbecca Moen, Barry S. Myers, Vonda Rodriquez, Tim Veldman, L. Ebony Boulware

**Affiliations:** 1 Duke University School of Medicine, Durham, NC, USA; 2 Duke Clinical Research Institute, Durham, NC, USA; 3 Duke Clinical and Translational Science Institute, Durham, NC, USA; 4 Duke Forge, Duke University School of Medicine, Durham, NC, USA; 5 Stanford University Department of Medicine, Stanford, CA, USA; 6 Verily Life Sciences, South San Francisco, CA, USA; 7 Center for Applied Genomics and Precision Medicine, Department of Medicine, Duke University, Durham, NC, USA; 8 Division of Cardiology, Department of Medicine, Duke University, Durham, NC, USA; 9 Department of Pediatrics, Duke University, Durham, NC, USA; 10 Department of Biomedical Engineering, Duke University, Durham, NC, USA; 11 Division of General Internal Medicine, Department of Medicine, Duke University, Durham, NC, USA

**Keywords:** Clinical trials, preclinical research, project management, translational science, CTSA

## Abstract

Project management expertise is employed across many professional sectors, including clinical research organizations, to ensure that efforts undertaken by the organization are completed on time and according to specifications and are capable of achieving the needed impact. Increasingly, project leaders (PLs) who possess this expertise are being employed in academic settings to support clinical and preclinical translational research team science. Duke University’s clinical and translational science enterprise has been an early adopter of project management to support clinical and preclinical programs. We review the history and evolution of project management and the PL role at Duke, examine case studies that illustrate their growing value to our academic research environment, and address challenges and solutions to employing project management in academia. Furthermore, we describe the critical role project leadership plays in accelerating and increasing the success of translational team science and team approaches frequently required for systems biology and “big data” scientific studies. Finally, we discuss perspectives from Duke project leadership professionals regarding the training needs and requirements for PLs working in academic clinical and translational science research settings.

## Background

The term *project management* describes a process whereby relevant knowledge, tools, and expertise are applied in a systematic, deliberate fashion to ensure that complex projects are successfully completed in a timely and efficient manner [1,2]. Evidence suggests that project management facilitates the delivery of project objectives and outcomes within the allotted time, budget, and resources [3]. Project management is widely applied across multiple sectors, including construction, manufacturing, the military, information systems, engineering, and government. It is particularly useful when experts from various disciplines or with varied expertise have to work in teams to accomplish specific goals [4,5].

Despite successful application in other fields, project management has been less widely adopted in academic science and clinical research, for reasons discussed below. However, the last two decades have seen increasing interest and investment in team science initiatives that leverage the strengths and expertise of professionals trained in different fields to work together to address scientific challenges [1,2,6–10]. This trend is exemplified by the 2016 enactment of the federal Program Management Improvement and Accountability Act [11], which encourages accountability and best practices in project and program management throughout the federal government. In addition, funding agencies, including the National Institutes of Health (NIH), increasingly stipulate effective project management plans as a mandatory element of funding proposals for studies involving highly complex interdisciplinary research (e.g., systems biology program grants).

We examine the roles and contributions of project management and project leaders (PLs) to academic science and clinical research. We also describe the specialized category of *project leadership*, a relatively new role that combines the organizational/operational skills typical of project management with the scientific and technical expertise needed to facilitate team science in the setting of translational research projects. We provide several illustrative examples of project leadership that draw upon our own recent institutional experience and offer suggestions for future directions in project management and leadership in academic research settings.

## Project Management in Research and Team Science

Although project management has widely been used in industrial settings (including pharma/biotech and other life sciences) to manage and achieve specific corporate goals, it is less common in academic research settings – a disparity that may reflect differences between industrial and academic environments. Industry research enterprises focus on translating products to market, with scientific research teams working toward company goals. Success is understood in terms of the alignment of deliverables with corporate goals and objectives, one of which is financial return on investment. In contrast, academic research enterprises tend to focus on creating and disseminating knowledge to society in general, and success is measured through the publication of generalizable research findings, the development of successful grant funding opportunities, and the perceived or measured impact of research on health or healthcare practice. As a result, areas of investigation among academic researchers are often broader than the targeted product areas pursued in industrial settings.

There is growing recognition in academia that complex collaborative research can benefit from a structured approach to translating the defined goals of the proposed scientific investigation [7]. This recognition is particularly pronounced in settings that have adopted team science approaches. The NIH and other funding agencies have prioritized team science as a means to accelerate translational discoveries, and the Clinical and Translational Science Award (CTSA) program in particular has been cited as an exemplary framework for developing multidisciplinary translational teams [12]. To date, however, little work has been done to describe the critical role performed by PLs in facilitating translational team science in academic settings or to share potential challenges and opportunities in leveraging PLs to achieve successful team science.

## Project Management versus Project Leadership

The project leadership role (see Panel) includes multiple functions and competencies that lie beyond the scope of traditional project management. PLs are best understood as nonfaculty operational partners or “extenders” of the principal investigator (PI) who are able to effectively bridge the scientific and operational domains. While the PI generates the scientific idea and provides key expertise in research design and data interpretation and the quantitative methodologist (e.g., biostatistician, epidemiologist, bioinformatician) designs the approach to collecting and analyzing data, the PL provides expertise in research operations and the management of team interactions. Importantly, PLs understand the scientific aspects of the project as well as the operational requirements (e.g., data management, regulatory, financial, vendor management, supply distribution). PLs draw on this unique combination of knowledge and skills to facilitate team science and ensure that the team functions effectively and efficiently throughout all stages of the project life cycle. The PI and PL meet with the initial team members to determine any additional expertise needed to ensure research success. The PL also conducts searches to identify internal or external expertise to add to the team, which may require, for example, identifying and evaluating vendors with appropriate expertise. The PL develops a well-defined project schedule incorporating dependency relationships and project deliverables from each team member. The PL has oversight responsibility to ensure that the work is completed as planned. In conjunction with the grants and contracts administrator, the PL also develops, negotiates, and oversees budgets and proposals. The PL also prepares narrative and analytical reports, documents, and correspondence for contracting agencies, team members, and senior management regarding study status. In summary, the PL provides day-to-day project oversight that allows the PI to focus on the scientific aspects of the research.

Panel Capabilities

- Substantial (e.g., 15+ years) research management (e.g., finance, regulatory) and coordination experience
- Advanced degree (e.g., PhD) in science-related field
- Experience with industry, government, other sponsors
- Detailed knowledge of the full translational science landscape (preclinical to population studies)
- Experience working with translational research engine (e.g., regulatory, technology transfer, methods experts, clinical trials) at Duke or elsewhere
- Trained in Team Science Leadership
- Demonstrates resilience, leadership, and actively facilitates change
- Communicates effectively with others, regardless of reporting relationship

Work Performed

- Coordinate complex initiatives
- Facilitate timely project benchmark achievement
- Anticipate and troubleshoot translation roadblocks
- Proactively connect teams with “translation facilitators” (e.g., regulatory or technology transfer specialists, and industry or venture capital)
- Lead and report on efforts to obtain and manage funding
- Frequently lead communications with other research groups or programs, serving as primary liaison and public relations lead for the research program
- Develop and coordinate wider program activities with responsibility for results in terms of costs, methods, and reporting requirements
- Develop strategies to improve or maintain the effectiveness of the research program
- Engage large groups of diverse stakeholders and facilitate the development of actionable plans and measurable outcome goals
- Manage progress of collaborating stakeholders toward achieving project goals
- Establish risk, cost, time, scope, communication, and quality management plans, update with input from stakeholders, and direct staff in execution of plans
- Provide significant intellectual contribution to the research program, including substantial leadership in developing the scientific content of research proposals and manuscripts
- Establish and maintain internal (Duke) and external communications to ensure successful research partnerships
- Frequently represent research program on behalf of the PI
- Provide guidance/mentorship as an operational subject matter expert to others who develop or manage large research programs or studies
- Oversee staff who manage the day-to-day activities for the research portfolio
- Independently write and edit significant sections of funding proposals and grants, reviews articles, synthesize the literature, and develop manuscripts
- Serve as an expert resource to faculty, trainees, and staff for the development of protocols for complex investigator-initiated studies
- Identify and assess issues and escalate to PI

**Specialized Capabilities of Duke Team Science Project Leaders and Key Team Contributions**

Team science is defined as a “collaborative effort to address a scientific challenge that leverages the strengths and expertise of professionals trained in different fields” [1]. Key elements of a successful team science approach include (1) a shared vision; (2) development of a shared vocabulary among team members; (3) explicit articulation of team member expectations, roles, and responsibilities; and (4) continuous communication across a complex landscape that encompasses scientific, regulatory, and commercial considerations [6]. It is the unique role of the PL to manage these elements. PLs support investigators by developing and executing these key elements of team science, both through coordination and by applying a large compendium of management tools. Management tools may include project charters, project development plans, role and responsibility grids, Gantt charts, communication and escalation plans, standard operating procedures, process flow diagrams, access to outside business experts, and expectation “contracts.”

PLs often have a diverse range of capabilities stemming from their educational focus, background, and experience, all of which may be beneficial when operationalizing a given project (Panel). The majority of PLs in the translational research domain have a clinical and/or scientific background, as well as qualifications and training reflected in degrees such as RN, PA, PharmD, or PhD (doctoral-level PLs are more common in early-phase translational research in which scientific and technical aspects of projects are critical factors). Some have obtained one or more certifications in project management, with the most common being the Project Management Professional (PMP) certification offered by the Project Management Institute [13].

PLs not only oversee project deliverables/timelines and budgets but also support the project’s strategic objectives and cultivate and manage interpersonal relationships among peers, collaborators, faculty, and sponsors [14]. In addition to demonstrating planning, prioritization, organizational, and time-management skills, successful PLs are also critical thinkers and problem solvers who apply communications skills to ensure team function. They demonstrate political awareness and sensitivity in building and managing professional relationships. As such, PLs must also be proficient in negotiation and conflict management, and they ideally have strong stress management skills. In industrial settings, PLs typically have authority and line responsibility that enables them to accomplish these goals. By contrast, academic PLs may play a supporting role to a faculty PI who has primary authority for the project. Hence, the skill set for an academic PL may need to include the ability to “manage up” [15] and be responsible with limited authority.

## Research Project Leadership at Duke University: A Case Study

Duke University has historically emphasized the integration of project management into the research workflow for studies conducted across the full spectrum of translational research. Duke’s early experience with logistical and organizational challenges that accompany large international cardiovascular clinical trials (“megatrials”) suggested that the conduct of clinical and translational team science benefits from the application of skills beyond those traditionally associated with clinical research study coordination (e.g., achieving regulatory or budget requirements for studies). This recognition of the need to more ably manage complex projects’ scope, timelines, and budgets led to the creation of a formal role centered on project leadership skills. PLs are equipped to execute all key elements (fostering shared vision, vocabulary, team member expectations, roles, and responsibilities, and continuous communication) to help complex projects achieve their visions.

PLs were integrated into the operations of the Duke Clinical Research Institute (DCRI), the university’s academic clinical research organization, in the mid-1990s. Since then, units across Duke have adopted and integrated the PL role within their organizations and management structures. Expanding on a model [16] used by for-profit contract research organizations and pharmaceutical companies, the DCRI embraced a “partner triad” leadership team comprising a PI, a PL, and a statistician. These triads, which emphasized collaboration and shared responsibilities for trial operations, were tasked with managing the megatrials for which the DCRI served as the coordinating center. As this model evolved over time, it was adapted to smaller multicenter trials across multiple therapeutic areas. Concurrently, PLs were recruited to support early-phase translational projects at Duke, consistent with the industrial model that applies project management in early-phase drug and device development projects.

### Applying the PL Model for Translational Studies in the Duke CTSA Program

Building on earlier models developed by the DCRI, the Duke CTSA Pilots Core (2013–2018) [17] sought to enhance and accelerate the scientific impact of translational studies supported by the CTSA. To accomplish this, the Duke CTSA cultivated a team of PLs to support the successful execution of translational studies by engaging team members from a broader range of backgrounds than those typical of interdisciplinary science teams. In addition to scientists, teams have included experts in regulatory science, reimbursement policies, market analysis, financial management, licensing, new venture creation, conflict-of-interest management, and navigation of sponsored research requirements such as contracts, institutional review board approval, and animal use committees. Each pilot study awarded through the Duke CTSA is required to incorporate PLs into the team structure and planning to ensure success. PLs have taken an active role to ensure teams work to ensure the maximum “translational capacity” of each awarded pilot program by ensuring that studies fully engage the expanded team of experts to achieve translational impact.

This newer Duke CTSA PL model has been well received by faculty members and has achieved substantial success. Faculty who work with PLs have recognized their value and have included them as team members on new sponsored research projects and applications. The model has also been recognized as innovative by members of the Duke CTSA External Advisory Committee, who have praised the model for ensuring “…access to a broad array of individuals with extremely valuable translational expertise that is rarely found in academic institutions, including legal and regulatory expertise,” noting also that the model could be adapted widely across different CTSA programs to “overcome barriers to translation” (Unpublished data, Duke University Clinical and Translational Science Award External Advisory Committee Report, 2008).

With PLs integrally involved in these faculty-initiated grants, the number of Duke CTSA team science PLs has grown to 10, with approximately 40 active projects under management. To date, team science PLs have helped the CTSA Pilots Core achieve multiple high-impact discoveries that together account for 21 filed patents, 11 invention disclosures, 15 new companies, and $133MM in follow-on funding across 78 total Pilots Core projects. The team science PLs also support the Duke-Coulter Translational Partnership, which has awarded $8MM for 39 projects, resulting in $486MM in follow-on funding, 13 licenses to industry, and 8 new companies. The team science PLs have also successfully facilitated population translation studies, including a regional study in which participants are providing genomic, metabolomic, physiologic, and behavioral data in a unique public–private partnership [18–20].

### Expanding a Culture of Team Science at Duke through PLs

The model of project leadership has spread across Duke’s research programs. The cross-campus diffusion of the PL model can be seen in multiple other Duke enterprises, as exemplified by the Center for Applied Genomics & Precision Medicine. The Center, which is focused on genomics discovery and innovation and implementation science, employs PLs through a unique functional matrix model in which they play integral roles at each step of the discovery and translational research process and partner with faculty to execute and manage both exploratory and clinical studies across the Center.

Of note, the Center is an outward-facing organization that seeks collaborative opportunities with research units across Duke, establishes industry partnerships, and pursues nontraditional funding mechanisms. Over the past decade, at least half of the Center’s external sponsor portfolio consists of US Department of Defense/military contracts. Such contracts typically support ambitious, time-intensive, high-risk research projects that demand exceptional performance and on-time deliverables. Being able to meet these demands within the traditional academic research, environment necessitated an evaluation of the underlying operational approach and implementation of effective project management structures and team strategies that undergird the Center’s model of operational excellence. Project management and the active involvement of PLs are essential parts of the Center’s fabric and facilitate the conduct of team science within academic and translational research environments.

Although use of the PL model is growing, PLs at Duke have traditionally operated in isolated pockets across the university and medical center. In order to provide training, promote intramural connections, share best practices, and encourage collaboration, the Clinical and Translational Science Institute, Duke’s CTSA-supported research entity, sponsored the Duke Project Management Community of Practice (PMCoP) to serve as a centralized hub that links a network of PL professionals supporting academic research throughout the institution [21]. This group launched in October 2017 and currently has more than 400 members representing the Schools of Medicine and Nursing, the College of Arts and Sciences, and the Duke University Health System. The Duke PMCoP surveyed its members in December 2017 in order to better characterize the community’s membership and current training and responsibilities. Table 1 shows the demographic features and experience of the PLs. In addition to enabling communication and networking across campus and beyond to other institutions and CTSA-supported programs, the Duke PMCoP fosters sharing and adoption of basic project management tools, procedures, processes, systems, and metrics. It also contributes to workforce development by identifying and providing educational and training opportunities for PLs across the institution, providing opportunities for professional development units required to support PMP certification and recertification, and supporting and participating in mentoring and internship programs. The Duke PMCoP has hosted more than 20 professional development events convened around a wide range of topics, including team science, implicit bias, critical soft skills for project managers, project management software tools, and leading virtual meetings.

**Table 1.** Survey results: characteristics of Duke project leaders (N = 67)

Characteristic	***N*** ** (%)**
Gender	
Male	7 (10)
Female	58 (87)
No response	2 (3)
Age in years	
25–34	10 (15)
35–44	30 (45)
45–54	20 (30)
>55	5 (7)
No response	2 (3)
Training	
RN diploma	1 (1.5)
BA/BS	20 (30)
MA, MS, MBA, or MPH	28 (42)
PhD	15 (22)
MD	2 (3)
No response	1 (1.5)
Formal project management training	
Yes	38 (57)
No	28 (42)
No response	1 (1)
Certification	
PMI PMP	12 (18)
No certification	55 (82)
Years of experience	
<1	7 (10)
1–5	20 (30)
5–10	22 (33)
10–20	15 (22)
> 20	3 (4)
Percentage of current job responsibilities involving project management	
90–100	32 (48)
50–85	28 (42)
10–50	7 (10)
Project management experience outside academia	
Yes	35 (52)
No	32 (48)
Type of project management experience outside academia (*n* = 35, *respondents were able to select more than one response*)	
Pharmaceutical/biotech	11 (31)
Healthcare	16 (46)
Information technology	10 (29)
Other	26 (74)
Experience managing team science projects	
Yes	50 (75)
No	11 (16)
Unsure	6 (9)

## Evolving Opportunities

Significant work remains in fostering a pervasive culture of project leadership across academia. Calls for the creation of the “interdisciplinary executive scientist” [22] highlight the need for individuals to serve in the project leadership role. A number of opportunities exist for PLs to facilitate this process in part by mitigating challenges to successful team science (Table 2).

**Table 2.** Enabling team science across disciplines: challenges and solutions

Issue	Solution	National implications
Translational barriers specific to each of the T1–T4 phases of the translational research process	Teams recruit PLs with specific translational expertise/skills; PLs identify collaborators as expert team members	PL roles become a requirement for translational research programs
Investigators/programs may have specific scientific needs but neither can support a full-time PL	Fractional PL support available from a central facilitative hub provides support as needed without extraneous overhead	Development of team science as the norm; acceleration of national translational science agenda
Team science increases complexity and communication requirements of research projects	PL can facilitate collaboration among members with multiple areas of expertise	Helps eliminate cultural/organizational barriers in evolution away from investigator-driven research and toward team science approaches
Investigators often must take on operational oversight and project management roles despite lack of training and skills	PLs are appropriately equipped to serve as operational leaders, so investigators can focus more on the science and research methods	Enhances project efficiency and reduces costs, with benefits to entire research enterprise
Assembling fundable proposals in big science, clinical and translational science	Institutional investment from indirect costs; federal investment specifying explicit PL requirements as part of proposals	Formalization of the role and requirements for project management as part of research
Lack of well-defined roles and funding	Integrate PLs into projects/programs in ways that empower them to contribute effectively, offer career incentives, and have clear lines of funding	Development of PM/PL as a discipline alongside the traditional research disciplines

PL, project leader; PM, project management.

### Enabling Team Science across Phases and Methodologies of Translational Studies

Team science PLs may be most successful when they focus their efforts to overcome translational barriers which are specific to studies within various phases of the translational science continuum, ranging from early lab-based discovery, to implementing discoveries in populations, to using “big data” methods to improve healthcare. For instance, translating a basic science discovery to a clinical setting (T1) may require expertise in areas such as animal safety and toxicology studies, identification of manufacturing facilities capable of producing a compound or device at the scale needed for animal and first-in-human studies, or in preparing regulatory submissions. Similarly, PLs moving a discovery from the laboratory into humans (T2) may need to facilitate collaborations with multiple clinicians and understand principles of pharmacokinetic/pharmacodynamic modeling in human research participants. Translating early-phase human research into clinical trials (T3) may require an understanding of clinical trial design, clinical endpoints, and differences between industry- and government-sponsored studies. Translation of evidence-based practices into real-world clinical practice and communities (T4) may involve additional in-depth expertise in implementation science and stakeholder, community, or policy engagement. Using large data sets to improve healthcare may necessitate basic familiarity with data structures, machine learning concepts, and other aspects of data science.

### Sharing Resources to Match Budget with Operations

Many individual investigators and organizational units could benefit from PLs, but they do not require or cannot afford a full-time PL to assist with a single investigator program. To provide a sustainable solution that enables multiple investigators to benefit, the Duke CTSA provides a coordinating service that provides fractional PL support to specific projects as needed, based on individual skill sets and the business, infrastructure, and scientific needs of each project. This approach ensures that operational work is performed by staff with the most appropriate skills, often at a fraction of the cost to the project that would otherwise be sustained. It also increases institutional funding of PLs, as units and unit-based grants are able to cost-share support for the CTSA PLs, resulting in substantially enhanced capacity and increasing the size and knowledge base of the institutional PL workforce. For instance, PLs are now supported in a wide range of Duke organizational units, including basic science departments in the Schools of Engineering and Medicine.

### Facilitating Internal and External Team Communication

Team science increases the complexity and communication requirements of research projects [23], while geographic dispersion may entail additional challenges. As noted above, translational research may require collaboration among team members with discipline-specific knowledge as well as expertise in statistical methodology, regulatory compliance, informatics, data management, stakeholder engagement, and finance. Given these disparate domains, strategic planning is critical for developing timelines, and constant vigilance is needed to ensure execution of deliverables. PLs could play an important role in establishing best practices for establishing effective communication among experts across disciplines and geographic locations.

### Helping Investigators Focus on Science

Researchers generally do not receive project management training and thus may be at a disadvantage when placed into team leadership or project management roles. Incorporating PLs into team leadership roles can allow scientific investigators to focus on their scientific and methodological areas of expertise, maximizing the possibility of project success.

### Risks and Limitations

Although we have listed multiple benefits that we have experienced with the PL model, a number of risks should also be considered and mitigated where possible. For instance, an effective PL model requires that scientific PIs and PLs have mutual respect for each other’s abilities and are able to work well together. However, academic research environments that emphasize hierarchical relationships and foster a high degree of autonomy for PIs can be challenging settings for establishing shared leadership models. PLs who are not part of a collaborative network that includes PIs or other team members may not be able to enact key determinants of their success (i.e., developing teams’ shared vision, vocabulary, and expectations). It is also important to consider how academic institutions can foster fulfilling career development and advancement opportunities for highly accomplished PLs to ensure that talented staffs are incentivized to remain engaged and that institutional knowledge and expertise are conserved. A proposal is under review to develop a university-wide, competency-based project leadership career ladder. This will need to be further supported by a more formalized management/leadership training program for persons interested in pursuing a career as a translational team science PL. Currently, the hiring and training of PLs is highly individualized according to the specific hiring unit and places substantial reliance on identifying candidates with experience in either a scientific discipline or project management role. “On the job” training and coaching are primarily used to develop the skill sets of new PLs. There may also be circumstances in which a PL role is not necessary, particularly in the case of programs focusing on potentially disruptive innovations [24]. As a recent analysis demonstrates, small teams focused on relatively high-risk projects may fare better without the structure and convention that a PL provides. Finally, although we have substantial anecdotal evidence that the PL model is beneficial to translational research at our own institution, we have not yet conducted empirical testing, which may offer future opportunities for learning as well as for generalizing our findings to other settings.

## Conclusions

Our experience at Duke suggests that team science is substantially facilitated by incorporating PLs who are trained to facilitate successful project and team science management into translational research teams. PIs who lead team science initiatives should embrace the PL model with the recognition that delegation and interdependence are key elements of team leadership. Academic institutions should also create training and clear career pathways with appropriate compensation for these PL roles. Research funding agencies should promote and fund the inclusion of PLs in team science initiatives. As other industries and our own experience at Duke demonstrate, the PL role is essential to successful teamwork. Given the educational background of Duke PLs, a training and career development program that can be integrated into more traditional academic career pathways can help to attract and retain experienced PL personnel. Finally, academic research institutions should consider deepening their institutional culture and commitment to PL engagement across the academic enterprise through adoption of this model.
